# Body, Soul and Spirit, an adaptation of two evidence-based interventions to promote physical activity and healthy eating among adults in churches in Lagos Nigeria: a three-arm cluster randomized controlled pilot trial

**DOI:** 10.1186/s40814-020-00600-6

**Published:** 2020-05-07

**Authors:** Oluwakemi Ololade Odukoya, Steve Manortey, Michelle Takemoto, Steve Alder, Kolawole S. Okuyemi

**Affiliations:** 1 Department of Community Health and Primary Care, College of Medicine, University of Lagos, State, Lagos, Nigeria; 2 Non-Communicable Disease Research Group, University of Lagos, State, Lagos, Nigeria; 3 Department of Family Medicine and Public Health, School of Medicine, UC San Diego, San Diego, CA USA; 4 ENSIGN School of Public Health, Kpong, Ghana; 5 Department of Family and Preventive Medicine, University of Utah School Of Medicine, Salt Lake City, UT USA

**Keywords:** Physical activity, Healthy food, Fruit and vegetable, Church, Cultural adaptation, Community-based

## Abstract

**Background:**

Physical inactivity and unhealthy eating are two leading behavioral risk factors contributing to preventable non-communicable diseases (NCDs). Evidence-based interventions (EBI) using community-engaged approaches to address these risks abound in high-income countries. Comparatively, evidence of such interventions is sparse in low- and middle-income countries, where NCD mortality is greater. This paper describes the protocol for the development of the cultural adaptation and pilot testing of a combination of two EBI (i.e., Body and Soul and the Healthy Body Healthy Spirit), in church-based settings in Lagos, Nigeria. In addition, we describe the development of the inclusion of an additional component, i.e., faith-based text messages, into one of the treatment arms. Our objective is to assess the feasibility of developing and implementing the adapted interventions with the ultimate aim of developing a fully powered trial.

**Methods:**

This pilot study will assess the design and implementation of a three-arm cluster-randomized pilot trial in 12 randomly selected Anglican churches (4 in each arm). First, we will design a cultural adaptation of the two EBI’s to form a multifaceted combined intervention known as the *Body Soul and Spirit*. The second treatment arm, i.e., *Body Soul and Spirit Plus*, will retain all the components of *Body Soul and Spirit* with the inclusion of faith-based text messages using mobile phones. Participants in the control arm will receive information leaflets designed to increase physical activity and healthy food consumption. The outcome measures include participant recruitment and retention, program participation and satisfaction, and data collection completion rates. The outcomes for the proposed definitive trial will be the number of servings of fruit and vegetables and minutes of moderate to vigorous physical activity per day will be assessed at baseline, 3 and 6-month follow-up. Implementation outcomes will be assessed using qualitative and quantitative methods.

**Discussion:**

The study will enhance the understanding of how best to design and implement behavioral interventions in church-based settings using community-based participatory approaches. It will also inform the development of a definitive randomized controlled trial.

**Trial registration:**

Pan African Clinical Trials Registry on 12th July 2018. PACTR201807136835945. Available at https://pactr.samrc.ac.za/TrialDisplay.aspx?TrialID=3481

## Background

Globally, unhealthy diets and physical inactivity are important risks for non-communicable diseases (NCDs) particularly cardiovascular diseases (CVD), type 2 diabetes, and certain cancers [1]. Up to 2.7 million deaths are attributable to diets low in fruits and vegetables. Worldwide, low intake of fruits and vegetables is estimated to cause about 19% of gastrointestinal cancer, about 31% of coronary heart disease, and 11% of stroke [1]. Almost two million deaths (1.9 million) are attributable to physical inactivity. Physical inactivity and unhealthy diets are major contributors to high body mass indices, which ranks as a leading risk of death globally [1, 2].

In many low- and middle-income countries (LMICs), the prevalence of these risk factors is rising, particularly among adults in urban areas [3, 4]. Several African countries are undergoing rapid urbanization and an epidemiological transition with increased rates of NCDs, particularly cardiovascular diseases, diabetes mellitus, cancers, and obesity [5–8]. In Nigeria, almost one in five deaths is attributable to NCDs, and this rate is increasing [9]. National surveys indicate that the rate of overweight/obesity among women increased from 22% in 2008 to 25% in 2013 with the highest prevalence (44%) occurring in Lagos, the most populous city in Africa [10].

Although several evidence-based interventions (EBI) that address known NCD risk factors exist, they are often generated in high-income countries (HIC) and are not frequently deployed in low- and middle-income countries where a significant burden of NCD mortality and ill-health occur [11–13]. There is limited evidence to demonstrate if or how EBI generated in HIC may work in low- and middle-income country (LMIC) settings with different religious, socio-economic, and cultural environments. Research that comprehensively addresses effective ways of adapting evidence-based interventions generated in HIC into local contexts can significantly advance the field of NCD prevention and result in significant improvements in population health outcomes [14].

The vast majority of people in many sub-Saharan African nations are deeply committed to the practices and major tenets of either Christianity or Islam, which are the world’s two largest religions. Almost all (> 95%) Nigerians identify with at least one of these two religions [10, 15]. Religious environments play an important role in promoting physical activity and healthy eating [16, 17]. Not only are they potential points of contact among the populace, they also tend to hold regular events that involve food, relationships, and other activities. Churches, in particular, present a potentially effective channel for delivering health programs [18, 19]. However, the majority of existing church-based trials have been conducted in HIC like the USA and among minority groups (e.g., African Americans or Latinos), and few have targeted multiple risk behaviors. Interventions targeting multiple behaviors simultaneously have the potential to be efficient, cost-effective, and produce meaningful changes in population health [20, 21].

Mobile phone use has increased tremendously in Nigeria over the past 15 years. Nigeria ranks second, only to South Africa, with the highest proportion of mobile phone users in Africa [22, 23]. In 2019, there were over 172 million mobile subscribers, accounting for an estimated 87% of the population. This figure represented a 6.4% growth increase, compared to 162 million subscribers in 2017 [24]. This provides a window of opportunity to develop mobile phone interventions to promote physical activity (PA) and healthy food consumption (HFC) as has been done in some HICs. Previous studies in HIC have used mobile phone interventions to promote these behaviors. Of the potential approaches to mobile phones interventions, text messages hold great promise, because they can be tailored, are relatively low cost and have the ability to reach a large proportion of people without the need for an internet connection [25, 26]. Although evidence shows that text messages are effective for improving PA and HFC in HIC [27–31], few interventions have considered the use of faith-based text messages to improve or sustain the effect of such interventions in faith-based settings. Furthermore, none of these studies have been conducted in a highly religious LMIC like Nigeria.

The aim of this study is to assess the feasibility of developing and implementing a cultural adaptation of the effective components of two evidence-based interventions in church settings, i.e., Body and Soul [32] and the Healthy Body Healthy Spirit [33], to increase PA and HFC in a church-based LMIC setting, with the ultimate aim of developing a fully powered trial. We also aim to assess the process of developing and implementing faith-based text messages as a component of one of the treatment arms.

### Theoretical framework

**Fig. 1:**
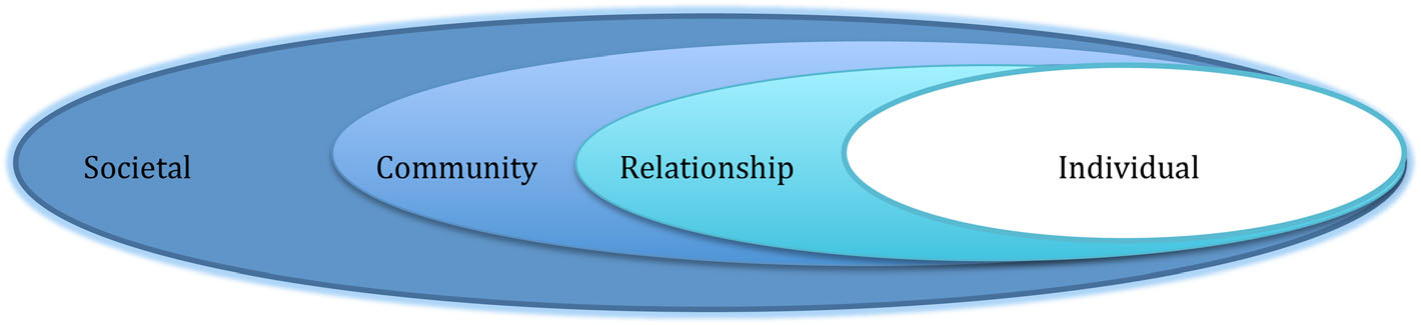
The socio-ecological theroretical model

As with the parent trials, we will be guided by the socio-ecologic theoretical model (Fig. 1) [34], which emphasizes the importance of the social environments that shape human behavior and addresses the personal, institutional, and community factors affecting them. The parent trials also emphasized the self-determination theory of motivation [35], which supports the intrinsic human tendency to behave in effective healthy ways.

A key aspect of this study involves the active participation of the community (church) members in its design and implementation. This is more likely to create sustainable interventions that promote community health [36]. In addition, this approach incorporates cultural norms and infuses community knowledge with evidence-based socio-behavioral theory for the effective implementation of complex health interventions [36, 37].

### Study objectives

1. Gather information from the church leadership and members and assess the process of using this information to develop a culturally adapted combined version of the two EBI and the additional faith-based text message component.
2. Explore the acceptability of each of the adapted components of the original interventions from the perspectives of the church leadership and church members and assess the feasibility of implementing each intervention component.
3. Assess the feasibility of recruitment and retention of the churches, church volunteers, and participants, assess consent rates and procedures, and gain estimates of recruitment and participation rates for the adapted interventions
4. Determine the acceptability, feasibility, and accuracy (and factors influencing this) of the data collection methods and measures, assess data completeness and explore data missingness at 3 and 6 months follow-up and its implications in developing a fully powered trial.
5. Assess the proportion of churches and study participants completing the culturally adapted intervention and explore factors that promote or discourage engagement with and completion of the intervention.
6. Estimate key parameters like the effect sizes, intra-class correlation coefficients (ICC), and standard deviations and compare these with the values obtained in the literature to inform the calculation of an adequate sample size for a fully powered definitive trial.

## Methods

### Study setting

This study will be carried out in Lagos State, the commercial capital and a largely urbanized part of Nigeria. It will be conducted among churches in the diocese of Lagos Mainland of the Anglican Communion, Church of Nigeria. The diocese is headed by a bishop and has a total of 53 churches. A vicar, assisted by church priests, is responsible for overseeing the administrative and religious activities of each church. Each church has a governing council consisting of elected members. Church services are held primarily on Sundays with other religious activities occurring on other days of the week as needed. Each church has a medical committee, consisting of volunteers with interests in health-related matters. In addition, there is a diocesan medical committee that oversees health-related church activities of the entire diocese.

### Trial design

This is a pilot of a three-arm cluster randomized controlled trial and will be conducted in 12 (4 in each group) of the 53 churches in the diocese of Lagos Mainland of the Anglican Church of Nigeria. The twelve churches will be randomized into three arms as follows:

A. Body Soul and Spirit (BSS)
B. Body Soul and Spirit Plus (BSS+)
C. Usual care/control (Control)

### Participants

The study will be conducted among members of the participating churches in the diocese.

### Inclusion and exclusion criteria

To be eligible, participants must be adults aged 18 years or older who have been members of the church for at least 6 months and attend church services at least weekly. Eligible respondents for BSS+ must, in addition, have a text message-compatible mobile phone and have used it for at least 6 months. Acutely ill or disabled people will be excluded.

### Description of the parent interventions

#### Body and Soul

This program [32] aimed at increasing fruit and vegetable consumption among African American church members living in California, Georgia, North Carolina, South Carolina, Delaware, and Virginia. The components included the formation of a church coordination committee, implementation of church-wide nutrition activities to increase fruit and vegetable intake, provision of educational materials, and motivational interviewing phone calls delivered by trained peer counselors and a policy change. A total of 15 churches were randomly assigned to the Body and Soul intervention or to a comparison group. Fruit and vegetable (F&V) intakes were measured at baseline and 6-month follow-up. Participants in the intervention group consumed significantly more F&V than in the comparison group [32].

#### Healthy Body/Healthy Spirit

This program [33] aimed at increasing physical activity and fruit and vegetable (F&V) consumption among African American church members living in the greater Atlanta area. Participants were provided with an exercise videotape and guidebook; a nutrition videotape, cookbook, and an audio cassette of gospel music. Sixteen churches were randomly assigned to three intervention conditions. Group 1 received culturally tailored self-help nutrition and physical activity intervention materials. Group 2 received the same intervention materials as group 1, plus four motivational interviewing phone calls delivered by trained peer counselors while group 3 served as the control group and received standard nutrition and physical activity materials. Physical activity and F&V consumption were measured at baseline and 1 year after. Participants in the intervention groups consumed significantly more F&V and exercised more minutes per week than those in the control churches [33].

### Description of the intervention arms

A. Body, Soul, and Spirit will be modeled after the two EBIs conducted in African American churches in the USA: the “Body and Soul” and the “Healthy Body Healthy Spirit” [32, 33]. The adapted intervention, Body, Soul, and Spirit, will involve a combination of the core components of both of the parent interventions. In creating the aggregate intervention, we will select components of each of the parent trials that were deemed “essential” [38, 39]. A component was termed “essential” if it was reported to have accounted for the positive intervention effects observed in the parent trials [40, 41]. The following components of the parent trials were considered essential and will be considered for our adaptation and use (see Table 1):

a) Individual education

i. An adapted version of a cookbook [32] with healthy eating recipes, cooking techniques, storage tips for fruits and vegetables, and health benefits of increasing fruit and vegetable intake.
ii. An adapted version of an exercise manual [33] and exercise video or audio CD [33] containing gospel music with biblical quotes/themes and/or brief sound bites of priest sermons sliced between songs.

b) Group education

Each participating church will receive a single copy of an adapted version of “Forgotten Miracles” [32] an 18-min video that targets fruit and vegetable intake using both spiritual and secular motivational messages. Churches will be asked to organize public screenings of the video and to make the copy available to members on request.

c) Campaigns and promotions

i. Formation of a project coordination committee: This committee will be composed of representatives of the academic and church communities and will be responsible for the implementation of the project [32, 33].
ii. Church events: There will be a project kick-off event, at least one nutrition event and an additional event involving the church priest [32]. Examples of events included in the parent trials are serving fruit and vegetables after church services, at other church programs, or during the kick-off event; sponsoring food demonstrations or taste tests; organizing tours of food markets and inviting guest speakers; or having priest sermons related to health (Table 1).

**Table 1:** Intervention components of the parent trials and adapted interventions

	Parent Trial		Adapted Intervention		
**Intervention Component**	**Body and Soul**	**Healthy Body Healthy Spirit**	**BSS (Arm 1)**	**BSS plus (Arm 2)**	**Control (Arm 3)**
A cookbook with healthy eating recipes, cooking techniques, storage tips for fruits and vegetables (F&V), and health benefits of lowering fat/increasing F&V intake	✔	✔	✔	✔	
An exercise manual with biblical themes and scripture woven throughout		✔	✔	✔	
A workout audio CD containing local gospel music with biblical quotes and brief sound bites of pastor sermons spliced between songs		✔	✔	✔	
Public screenings of “Forgotten Miracles,” a video that targets fruit and vegetable intake using both spiritual and secular motivational messages	✔	✔	✔	✔	
Constitution of a project coordination committee	✔	✔	✔	✔	
Conduct at least one health event*	✔		✔	✔	
Motivational interviewing phone calls delivered to participants by trained volunteer peer counselors	✔	✔	✔	✔	
Churches will agree to implement at least one policy change^#^	✔		✔	✔	
Faith-based text messages				✔	
Distribution of information leaflets promoting physical activity and healthy food consumption			✔	✔	✔

*Examples of some of the events included in the parent trials were serving fruit and vegetables during the kick-off event, after church services, or at other church programs; sponsoring food demonstrations or taste tests; organizing tours of food markets for parishioners; inviting guest speakers; and having priest sermons related to health

^#^Potential policy changes include establishing guidelines for the types of foods served at church functions, changing snacks served at youth camps, or creating a food pantry

d) Supportive relationships

Motivational interviewing phone calls to selected church parishioners delivered by trained church volunteers [32, 33].

e) Policy change

Churches will agree to implement at least one policy change [32]. Potential policy changes include establishing guidelines for the types of foods served at church functions, changing snacks served at youth camps, or creating a food pantry.

B. Body Soul and Spirit Plus (Bss+) will retain all the components of BSS with the addition of faith-based text messages. We will develop the content, wording, frequency, and timing of each message guided by the theoretical models mentioned above, a review of existing literature and the feedback received from the parishioners. We will then pre-test them among a small group of parishioners before use to ensure that the messages are designed to have the desired effect. The project coordinator and/or trained church volunteers will send these messages periodically at pre-scheduled intervals.

**Fig. 2:**
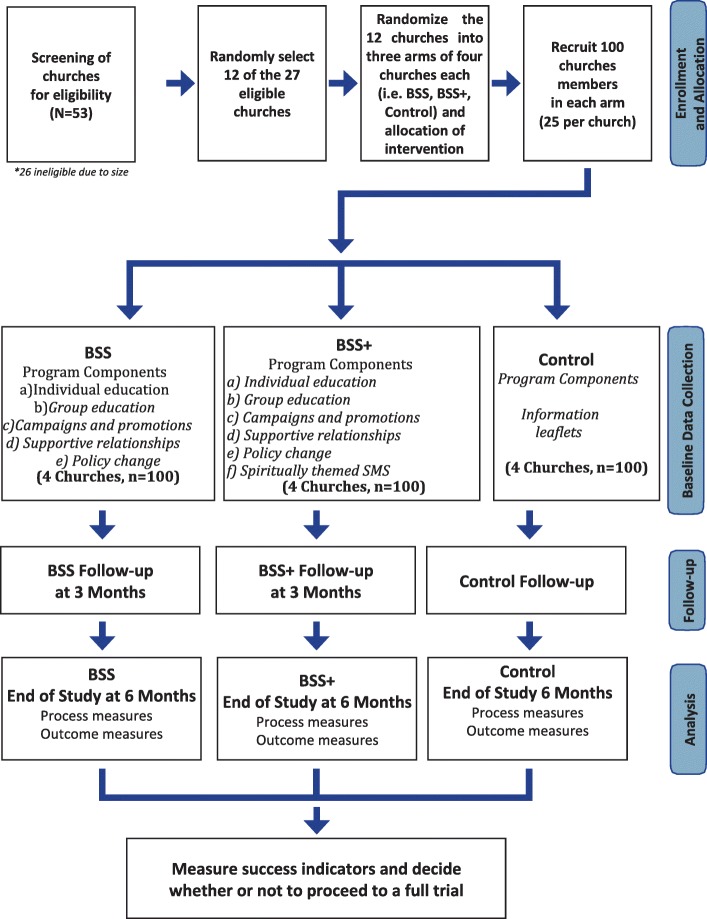
Flow chart for participant enrolment, baseline data collection, follow-up, and analysis

C. Usual care/Control: Information leaflets on healthy food consumption and physical activity will be distributed to parishioners in the churches in this group after a church service. This will constitute an information-based *standard of care* approach that will be used as the control condition for this group randomized trial (See Fig. 2).

### Phases of the study

The study will be conducted in four phases.

*Phase 1—*Pre-implementation—Community entry, recruitment, and randomization of churches; partnership and capacity building; and needs assessment and situation analysis.

*Phase 2*—Intervention adaptation and development—Adaptation and design of the core components of BSS and BSS+ for use in a Nigerian setting.

*Phase 3*—Intervention implementation and delivery

*Phase 4*—Implementation evaluation—Data collection and analysis of implementation measures, study outcomes, and success criteria; calculation of an ICC, the means and variances for each the three treatment arms, effect sizes (all to potentially inform the sample size calculation in a full RCT), and the decision to determine whether or not to progress to a definitive trial.

#### Phase 1—pre-implementation

*i*) *Community entry, church recruitment and randomization:* We will work with the leadership of the diocese to recruit the 12 eligible churches from their network of churches. To be included in the study, churches must have at least 100 active members (we define “active” as members who attend church services at least weekly). We will obtain a list of the churches in the diocese and screen them for eligibility. Then, we will randomly select 12 eligible churches from this list. Selected churches will be allocated into one of three groups: BSS, BSS+, or control. Randomization will be computer-generated, with allocation concealment by opaque sequentially numbered sealed envelopes. An independent biostatistician will generate the randomization sequence in accordance with the CONSORT guidelines [42].

*ii*) *Partnership development and Capacity building:* We will create a diocesan-project steering committee (DPSC) consisting of one representative from the diocesan leadership, one representative of the diocesan medical society, two diocesan volunteers, and members of the academic research team. The DPSC will serve as the overall planning and coordination team for the intervention. In addition, in each of the eight intervention churches, we will create a local church implementing committee (LCIC), which will consist of at least two church members who are either volunteers and/or persons nominated by the vicar and members of the academic research team. The LCIC will serve as the in-house implementation team for the intervention.

Once the committees have been constituted, we will conduct a partnership development and capacity building workshop for committee members to obtain leadership and community buy-in. Workshops will initially focus on sharing the project aims with participants to obtain their support and commitment and to engage them as partners on the project. The committees will hold regular meetings where the academic researchers will work with them to identify effective ways of implementing the intervention. Thereafter, the committee members will meet at regular intervals to discuss the development, planning, and implementation of the project. Committees will decide on operational procedures and develop a plan to keep their church parishioners involved in the intervention.

*iii*) *Needs Assessment & Baseline Situation Analysis:* Qualitative methods will be used to: (i) Identify and understand the underlying factors influencing PA and eating patterns of the parishioners. (ii) Explore their underlying cultural and spiritual values/norms and how these affect PA and HFC. (iii) Explore their attitudes towards the faith-based text messages and seek their opinions on best ways to engage parishioners using such messages. (iv) Understand how best to adapt the core components of the program in their local setting.

We will conduct nine in-depth interviews with the bishop or his representative (1), church leaders/priests/members of the church governing council (5), and members of church medical societies (3). We will conduct focus group discussions (FGDs) with the church members. Participants will be stratified into three distinct groups according to their ages, i.e., the elderly > 65 years, adults 25–64 years, youth aged 18–24 years, or persons who attend the youth section of the church. Focus groups will consist of 6–12 participants per group. All interviews and FGDs will be conducted in the preferred language and setting of the participants, audio-recorded, and a research assistant will take notes during each discussion/interview.

#### Phase 2: intervention adaptation and development—adaptation of the core components of BSS and BSS+ for use in a Nigerian setting

Adaptation involves “reviewing and changing the structure of a program or practice to more appropriately fit the needs and preferences of a particular group or community” [43]. To culturally adapt the core components of the project, we will go through the five stages of cultural adaptation proposed by Barrera et al., i.e., information gathering, preliminary design, preliminary testing, refinement, and final trial [44].

*Information gathering*: First, we will gather information to determine how well the original intervention will fit the needs and preferences of our population, and if an adaptation is in fact justified.

*Preliminary design*: We will use the information gained from the first stage to inform the preliminary modifications of the original interventions while attempting to preserve the core components. This will be done using an iterative process in a series of focus group discussions and feedback sessions with the church committee members employing the same qualitative methods described earlier. Thereafter, we will draft the preliminary version of the intervention.

*Preliminary testing, refinement, and final trial*: This study is the focus of the third and fourth stages where we aim to pilot test the preliminary version and refine it with the ultimate aim of proceeding to Barrera’s fifth stage of testing the interventions in a fully powered trial.

##### Framework for program adaptation and balancing fidelity and fit

Bernal et al. [45] proposed one of the earliest models, the ecological validity model (EVM), for cultural adaptation of evidence-based interventions. This delineates eight dimensions, which we will consider when developing the cultural adaptation of the program. They are language, persons, metaphors, content, concepts, goals, methods, and context [38]. Rodriguez et al. [46] later expatiated on this to include three phases of adaptation. In the initial phase, the research team will collaborate with the members of the DPSC to find a balance between the needs of the church members and scientific integrity. Then, we will jointly identify relevant evaluation measures in the parent trials and adapt them in a process parallel to the adaptation of the intervention. The final phase will consist of jointly integrating the observations and data gathered to design the newly packaged intervention [46–48]. This process will also follow the guide for balancing program fidelity and fit as proposed by Castro et al. [49] during the program adaptation process.

In a series of workshops, the DPSC, guided by the core components of the BSS and BSS+, the results of the needs assessments, focus groups, feedback sessions, and the models described above, will carry out the project adaptation process. They will review the study protocol and intervention materials, consider session-by-session analysis of each component and recommend possible modifications to ensure the spiritual and cultural acceptability to the parishioners. Based on the recommendations of the workshop participants, modified intervention materials will be created. The end-result will form the initial draft of the Body, Soul, and Spirit program.

##### Development of the faith-based text messages for BSS+

The diocesan project steering committee and the members of the academic team will jointly develop a series of faith-based text messages designed to promote PA and HFC for church members. We will design the messages considering the World Health Organization (WHO) recommendations for physical activity and healthy eating and using the guidelines proposed by Abroms et al. [50] for developing and pretesting text messages for behavioral change, which are as follows:

a) Conduct formative research for insights into church members’ health behaviors and opinions of faith-based text messages to promote PA and F&V intake: In a series of focus group sessions with the project committee and church members, we will explore the opinions of the church members about physical activity and fruit and vegetable consumption, the possible barriers to these health behaviors, and their opinions about receiving text messages to help them overcome these barriers.

b) Design the text messages (TM): Then, we will identify our key behavioral change goals. Our objectives for the TM component are to provide knowledge and/or fill in identified knowledge gaps, modify attitudes, address known barriers to change, and motivate people to take action towards our key study outcomes. A key consideration in designing the faith-based messages is to establish the link between biblical themes and our behavioral change goals. Guided by our communication objectives, theoretical models and using the insights garnered from the focus groups and the literature, we will develop the TM framework (i.e., frequency, timing, wording, language) and form the faith-based TM library. This will be done using an iterative process of feedback sessions with the church committee members.

c) Pretesting and revising the text messages: We will identify a small set of church members (less than 30) in similar churches that are not part of our study. We will pre-test these messages using the agreed TM framework, solicit the feedback from the participants on the wording, content, and perceived effect of the messages. Thereafter, we will make appropriate modifications till a final version of the TM library and framework is agreed upon. Then, we will adopt this for use as the **+**PLUS component of the BSS**+** intervention arm.

Finally, based on the drafts developed for the BSS and BSS+, the DPSC in another series of workshops, will develop an action plan for the programs and present this plan to the Bishop or his representative for his input prior to adoption. These will serve as the action plans for BSS and BSS+ programs.

#### Phase 3: intervention delivery/implementation

We will employ a three-arm group randomized pilot study design using 12 churches (4 in each group). The intervention will operate at two levels. The church-wide events and environmental changes will be aimed at the entire congregation, while the distribution of exercise/nutrition videos, manuals and cookbook recipes, audio CDs, and text messages will be delivered only to individuals who voluntarily enroll and consent to participate in the study. Data will be collected at baseline 3 and 6-month time points.

Each participating church will identify 2–3 volunteers to serve as peer counselors for this project. They will participate in the recruitment of study participants and undergo peer-counselling training for the program. Volunteers will have a background in a “health-related profession” (e.g., physician, nurse, or other health worker) and will be required to attend a training, make four intervention calls to at least five members of each church, and undergo a performance evaluation before engagement. This training will provide knowledge and general skills in patient recruitment, research ethics, and motivational interviewing techniques like asking open-ended questions, reflective listening, and specific strategies to elicit discussions about PA and HFC. They will be asked to recruit and make at least four intervention calls to at least five study participants. Peer counselors will receive a small stipend for their time and effort with participant recruitment and retention and calling cards to cover the cost of the phone calls. In addition, regardless of the treatment arm, churches (rather than participants) that achieve the minimum targets for participant recruitment and retention will be provided with an incentive worth $100 or less at the end of the study.

#### Phase 4

Implementation evaluation—This will involve quantitative and qualitative data collection and analysis of implementation measures (i.e., participant recruitment and retention; Program participation and satisfaction and data collection completion), the calculation of parameter estimates for subsequent sample size calculations for the future trial, evaluation of the program success criteria, and a decision to determine whether or not to progress to a definitive trial.

### Outcome measures

The outcome measures for the pilot trial include the following:

1. Recruitment measures: We will calculate the number of participants who respond to the research call and are screened for eligibility, number/proportion of screened participants who meet the criteria for eligibility, number/proportion of eligible participants who provide consent and are enrolled in the study, average time taken to enroll the required number of study participants, and reasons for any delays, reasons for non-consent, and primary reasons for non-eligibility.
2. Study implementation and fidelity measures are as follows:

- *Individual and education:* Number of church members who request copies of the study materials. Proportion of enrolled church members who receive the cookbooks/exercise manuals/CDs, admit to using them, how often they are used, the self-rated usefulness of the materials in relation to study outcomes, and reasons for non-use.
- *Church committees and group events*: Number of nominated committee members who consent to participate in the church project committees, number of consenting members who remain as members throughout the study period, attendance at church events, and rated usefulness of project events in relation to trial outcomes.
- *Peer Counselors*: Number of church members who volunteer to serve as peer counselors, proportion of potential peer counselors who attend the training, proportion of trained MI counselors who satisfy the post-training evaluation, and proportion of trained peer counselors who remain in the program.
- *Church policy:* We will assess the perception of the selected church policies and perceived level of policy compliance.
- *Faith based text messages*: We will calculate the proportion of study participants who received, opened, and read the messages; participants’ ratings of messages in relation to trial outcomes.
- *Overall study fidelity of intervention delivery*: This will be assessed based on the delivery of each of the intervention components as stated in the study protocol and action plan. We will measure this using a structured checklist by direct observation of the implementation of each of the intervention components, noting any deviations from protocol/action plan.

3. Retention measures: This will be calculated as the proportion of committee members, peer counselors, and enrolled study participants who remain in the study at 3 and 6 months. We will also assess reasons for attrition among the study drop-outs.
4. Data collection measures: We will assess the proportion of completed surveys, time taken, and number of visits/phone calls/reminders required to complete surveys, levels of missing data, and reasons for incomplete surveys and variable missingness.
5. Data required for sample size estimations for a future definitive trial: These will be measured by computing the intracluster correlation coefficient (ICC), effect size estimates, variances, and 95% confidence intervals for the potential differences between the study arms at baseline and follow-up.
6. Acceptability and program satisfaction measures: We will measure program satisfaction using a self-reported rating among study participants. This will be done for each of the components of and the overall adapted intervention.
7. Measures for the end-points and psychological mediators for the proposed definitive trial: We will also collect data on the primary and secondary end-points for the proposed definitive trial and assess the process of collecting this data. In the parent trials, the primary outcomes were the number of servings of fruit and vegetables (F&V) and the minutes of moderate to vigorous PA per day (MVPA). The secondary outcomes included food consumption, vegetable preparation practices, intrinsic/extrinsic motivation, and self-efficacy to eat F&V and to increase MVPA. The main trials also assessed psychological mediators like physical well-being, depression, and religiosity indices. As with the parent trials, we will use a modified version of the National Cancer Institute (NCI) F&V food frequency questionnaire to assess food intake over the past month [51–53]. Vegetable preparation practices, self-efficacy, and other health habits will be assessed with an adapted version of the instruments developed for the eat for life program [54]. Intrinsic/extrinsic motivation will be assessed with an adapted version of the treatment self-regulation questionnaire (TSRQ) measure [55]. Physical activity and sedentary behavior will be assessed using adapted versions of the International Physical Activity Questionnaire (IPAQ) and WHO STEPS tools [56, 57]. Psychological mediators like physical well-being will be measured using the widely used single item measure on self-health [58]. Depression will be assessed using the patient health questionnaire-9, previously validated for use in this setting [59, 60], while religiosity and organized religious activity will be measured with two variables, church attendance, and private prayer frequency [61].

### Sample size estimation

In a pilot trial, the objective is not necessarily to prove superiority of the treatment but to test trial procedures and processes and to obtain estimates of parameters for the main trial sample size calculation. Therefore, standard sample size formulae which are used for main treatment assessments are not usually applicable to pilot trials [62, 63] see Additional file 1. As recommended in the CONSORT guidelines for publishing protocols of pilot studies [62], we made a pragmatic decision to select 12 churches for the study and 25 participants per church based on our knowledge of the church structure, its demographics, and our pilot objectives.

### Recruitment and retention strategies

Churches will make announcements for participation in the project, during church services, and using bulletin inserts or other usual means of communication. Trained church volunteers and/or research assistants will help to recruit interested participants. This will be based on participant eligibility and on a first-come, first-served basis. Recruited participants from each church will be selected to participate in the individual program activities and complete the surveys. Participant recruitment will be conducted in the three study arms in the same manner regardless of treatment allocation.

To promote participant retention, we will provide a small incentive (a gift worth $5 or less) to all participants regardless of the treatment arm. In addition, participants will receive at least two text messages and one phone call reminder to schedule a date for the follow-up surveys. We expect to be able to complete participant enrolment over a period of 12 weeks and will aim for at least 80% retention at the 6-month follow-up, noting any differences in attrition rates between study groups.

### Data collection

Study data will be collected at three-time points (at baseline, then 3 and 6 months after) (Table 2). The adapted questionnaires will be completed in the church premises either before or after a routine church program, or as is convenient for the participant. Participants will have the choice of filling in questionnaires by self-report or with the assistance of a trained interviewer and in their preferred language. Survey tools will be translated and back-translated into the preferred language of the parishioner if the need arises. Participants not responding to the follow-up questionnaires will be contacted on the telephone by the trained interviewers and offered to complete the instrument either by a scheduled home visit or over the phone. We will analyse participants’ responses for those completing the instruments by self-report, with the help of trained interviewers and by phone to observe for any statistically significant differences. At the end of each data collection session, we will ask the participants to rate their level of satisfaction with the data collection process. We will also note the time taken and number of visits required to complete each data collection session. At the end of the study, participants will fill a brief survey to assess their self-reported exposure, overall program satisfaction, and individual satisfaction with each of the program components. Figure 2 shows the study flow chart for participant enrolment, baseline data collection, follow-up, and analysis & Table 2 shows schedule of enrolment, interventions and assesements

**Table 2:**
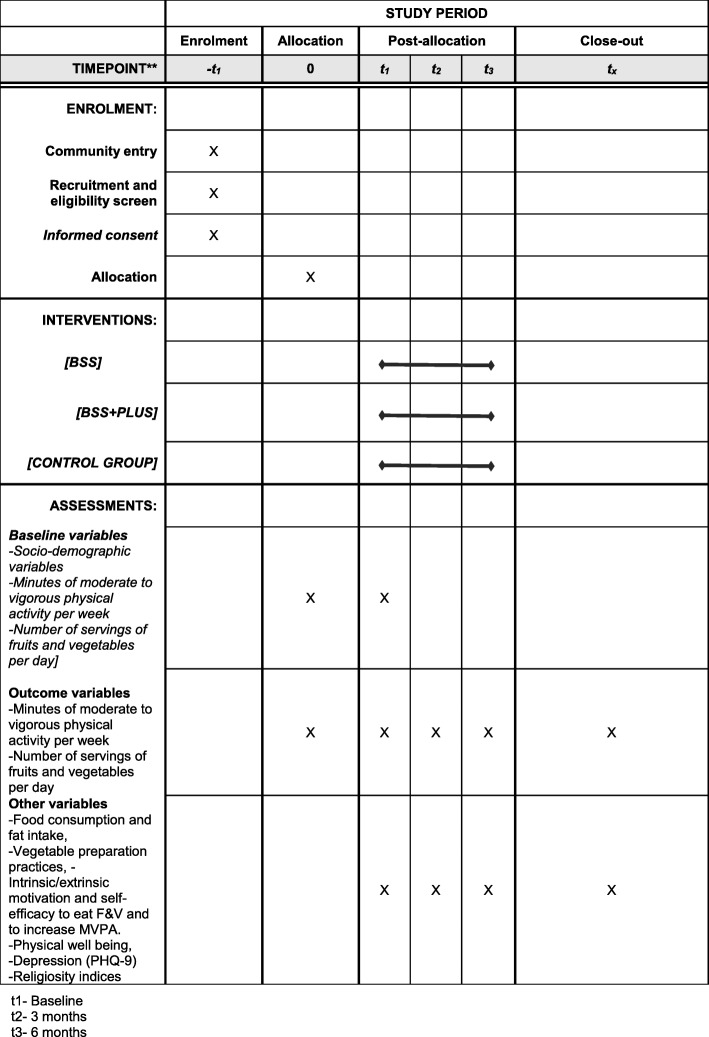
Schedule of enrolment, interventions, and assessments

*t*_1_ baseline, *t*_2_ 3 months, *t*_3_ 6 months

We will also conduct a series of focus group sessions to qualitatively assess the implementation measures. We will hold sessions with the study participants to obtain their feedback about their opinions and experiences of the BSS and BSS+ programs and processes, including the facilitators and barriers to participant retention. We will collect information on participants’ experiences of the intervention and its components challenges with completing questionnaires, their concerns about the quality and delivery of each of the intervention components, and any potential impediments to the successful initiation of a definitive trial. We will conduct additional focus group discussions with the data collectors, peer counselors, and the members of the steering committees to obtain their feedback of the study processes, experiences with the program, and the challenges they may have experienced with adhering to the protocol.

### Data analysis:

#### Quantitative data analyses

As this is a pilot study, significance tests of change over time will not be performed on the primary or secondary outcomes. The main analysis will focus on descriptive statistics relating to feasibility to estimate likely recruitment, retention, and participation rates. These will be represented by point estimates and 95% confidence intervals. We will compute the intra class correlation coefficient, effect size, and variance and use these in addition to estimates derived from the literature to estimate a sample size required for a definitive trial, if deemed appropriate. Quantitative data will be analyzed using STATA 15.0

#### Success indicators

The rationale for the success indicators was based on the process values obtained in one of the parent trials, where the process trial yielded 65.8% member participation and 100% church participation rates [64]. The success indicators for this pilot feasibility trial would be to:

1. Successfully develop a culturally adapted version of the two RTI as evidenced by a clearly defined BSS and BSS^+^ action plan approved by the church leadership and committee members.
2. Achieve the 70% recruitment targets for churches, church volunteers, and study participants and the time used to meet these targets.
3. Successfully deliver the adapted intervention to at least 70% of the selected churches.
4. Church compliance with at least 70% of the intervention components and respondent participation in at least 70% of the components.
5. Church retention rate of 100% at 6-month follow-up and at 70% or more for church committee members, volunteers, and study participants at 3 and 6 months.
6. Data completion rates and data completeness of ≥ 70% for each outcome measure and data collection method.

#### Qualitative data analyses

Audio-recorded interviews and focus groups will be transcribed verbatim, and we will perform a content analysis using standard thematic analytic techniques. At least two independent researchers will code each transcript before subsequent summarization and interpretation. Data will be analyzed with the Dedoose 8.18. software [65].

Collectively, these will provide information on the feasibility of the intervention implementation, trial procedures, data collection methods, and the overall acceptability of the intervention. The achievement of the success criteria stated above, in addition to the qualitative information generated from this pilot, will be considered in deciding whether or not to proceed with the intervention, with or without amendments. We will note which amendments to consider in developing a refined version of the intervention protocol for a fully powered trial to measure the effect of BSS and BSS+ on physical activity and fruit and vegetable consumption among church members.

## Discussion

The described study protocol is intended to pilot-test the feasibility and initial effects of the cultural adaptation of two evidence-based interventions in church settings using a 3-arm cluster RCT. The selected studies are a part of National Cancer Institute research-tested intervention programs (NCI-RTIPs) [11]. This is a database of evidence-based cancer control programs, which have been tested in a research study and have findings that produced one or more positive behavioral and/or psychosocial outcomes (*p* ≤ .05) among individuals, communities, or populations. To be listed on the NCI-RTIPs database, evidence of study outcomes must have been demonstrated using an experimental or quasi-experimental study design [10]. We selected the Body and Soul [32] and Health Body Healthy Spirit [33] because both programs were conducted in church-based settings and among persons of African heritage. Body and Soul focused primarily on improving fruit and vegetable intake while HBHS focused on increasing physical activity. The adapted intervention will focus on both healthy eating and physical activity, as these behaviors are often interrelated, and a combined intervention may have a synergistic effect, compared with stand-alone programs, which focus on only one behavioral outcome [20, 21].

The inclusion of the second intervention arm (i.e., BSS+) of the RCT was based on the premise that Nigeria as with many LMICs have a rising proportion of mobile phone users. We, therefore, sought to use this as a leverage and included a “plus” component into this study, to determine if faith-based SMS prompters are feasible and may be used to improve or sustain the effects of the adapted intervention. The components of the BSS and BSS plus have actions targeted at the individual and community levels. This is in line with the recommendations of Coughlin et al. for multi-level interventions, in their review of community-based studies targeting healthy diet, nutrition, and weight management among African Americans [66]. Many of the studies identified in that review showed that community members are often quite interested in community-based research and are willing to work with researchers to ensure that the interventions developed are tailored to the needs of their community [67]. Ours is one of the first few studies in sub-Saharan Africa that employs a partnership between an academic institution and a church community to address healthy diet and physical activity. Evidence generated from studies that involve partnerships between academic institutions and community partners has the potential to contribute new, scalable, and sustainable findings to address key NCD risk factors within local communities [66, 67].

.

We expect that this pilot study will provide the evidence base for the design, adaptation, and implementation of programs promoting PA and HFC in faith-based settings in highly religious low- and middle-income communities. The lessons learned would be useful in adapting such EBI’s to other faith-based settings, such as those of the Islamic faith and to workplaces and other community-based settings, to extend the impact of research-tested interventions beyond the settings in which they were developed. If the inclusion of the text message prompters is found to increase the effectiveness of these interventions, then there is the potential to reach and influence large numbers of people at relatively low cost. This holds great value for implementation and sustainability in many resource-constrained countries.

### Study limitation

As with other community-engaged health interventions, one possible limitation that could occur is in balancing fidelity and fit. This is because community participants may have their own ideas about how the intervention should be implemented, and this may be at variance with our study protocol. We will therefore employ Castro et al.’s [49] recommendations on balancing fidelity and fit as we implement this trial. We will also document any variations between the proposed trial and the implemented version.

### Dissemination plan

Project findings will be disseminated to the health committees and church members at the diocese and in the participating churches. Brief reports will be sent to the community engagement/non-communicable disease units of the state and federal ministries of health. Reports will also be sent to local media.

## Supplementary information

**Additional file 1.** Appendix 1 Sample size calculation.

## Data Availability

Not applicable

## Abbreviations

BS: Body and Soul
BSS: Body Soul and Spirit
CONSORT: Consolidated Standards of Reporting Trials
DPSC: Diocesan project steering committee
DSMB: Data Safety Management Board
EVM: Ecological validity model
FGD: Focus group discussion
F&V: Fruit and vegetable
HBHS: Healthy Body Healthy Spirit
HFC: Healthy food consumption
HIC: High-income country
IPAQ: International Physical Activity Questionnaire
LCIC: Local church implementing committee
LMIC: Low- and middle-income country
MVPA: Moderate-vigorous physical activity
NCD: Non-communicable diseases
NCI: National Cancer Institute
PA: Physical activity
WHO: World Health Organization
WHO STEPS: World Health Organization Stepwise Approach to Surveillance
