# Supplementary material for: Body, Soul and Spirit, an adaptation of two evidence-based interventions to promote physical activity and healthy eating among adults in churches in Lagos Nigeria: a three-arm cluster randomized controlled pilot trial
Source: Pilot Feasibility Stud. 2020 May 7;6:59. doi: 10.1186/s40814-020-00600-6 (PMC7203804; doi:10.1186/s40814-020-00600-6)
Supplement: Supplementary file 1 — Additional file 1. Appendix 1 Sample size calculation. [file 40814_2020_600_MOESM1_ESM.docx]

**Appendix 1 Sample size calculation**

To calculate the sample size for the definitive trial, we used the online Cluster/Group Randomized Trial Sample Size Calculator developed by the National Institutes for Health (NIH) ^1^ This allows us to impute a set of variables (the level of significance, study power, ICC, expected effect sizes and variance) and use these values to compute the minimum number of clusters required for an appropriately powered definitive trial.

Hayes and Moulton’s recommend separate sample size calculations for 3-arm RCTs.^2^ In computing our figures, we therefore considered two scenarios. First, to estimate differences between the intervention and control arms (either BSS or BSS+ vs. Control) and then, between both intervention arms. (BSS and BSS+)

Furthermore, since the proposed definitive trial has two primary outcomes, we calculated cluster sizes separately for each of the primary outcome variables (minutes of moderate to vigorous physical activity per day and number of daily servings of fruits and vegetables).

In the first scenario, we used a 5% level of significance, 80% power, estimated ICC of 0.02, an expected 20% difference in study outcomes and a pragmatically chosen number of 25 respondents per cluster. In the second scenario, we used the same parameters stated above, but adjusted our expected effect size to a 10% expected difference in study outcomes between the two treatment arms. (BSS and BSS+)

In both scenarios, as reported in previous studies,^3,4^ we used baseline values, (i.e. mean(SD) for each of the primary outcomes) of 63.4(37.4) and 4.0(2.5) for MVPA and the number of servings of daily servings of fruits and vegetables respectively. Thus, values of 1398.8^3^ and 6.25^4^ were used as estimates for the variance of both outcomes

Based on these imputations, we obtained a minimum cluster size of 10 & 11 (treatment vs. Control) and 34 & 38 (between treatment arms) for MVPA and number of daily servings of fruits and vegetables respectively. Considering the largest number, the sample size for a fully powered study would be 38 clusters. See Appendix 1.

However, since this is a pilot study, the CONSORT guidelines state that the objective is not necessarily to prove effectiveness but to test trial processes and procedures,^5^ so, based on our experience of the church structure, its demographics and study feasibility objectives, we have pragmatically selected a sample size of 4 clusters per treatment arm and 25 respondents per cluster for this study. Hayes and Moulton also recommend this as a minimum rule of thumb for cluster sizes. Our analyses are mainly descriptive and we expect that we will be able to sufficiently address our pilot objectives with this sample. We remain aware that we will not be testing for statistically significant differences in treatment outcomes in this pilot, as it is underpowered to do so.

Below are different scenarios of cluster size calculations based on ICC of 0.02 and 0.05.

| ICC**^@^** | Mean (SD)^♯^ | % change in effect size | Primary outcome**^^^** | Minimum number of clusters required |
| --- | --- | --- | --- | --- |
| 0.02 | 63.4±37.4**^¢^** | 20**^*^** | MVPA | 10 |
| 0.02 | 63.4±37.4**^¢^** | 10**^£^** | MVPA | 34 |
| 0.05 | 63.4±37.4**^¢^** | 20**^*^** | MVPA | 14 |
| 0.05 | 63.4±37.4**^¢^** | 10**^£^** | MVPA | 50 |
| 0.02 | 4.0±2.5**^§^** | 20**^*^** | NSF&V | 11 |
| 0.02 | 4.0±2.5**^§^** | 10**^£^** | NSF&V | 38 |
| 0.05 | 4.0±2.5**^§^** | 20**^*^** | NSF&V | 15 |
| 0.05 | 4.0±2.5**^§^** | 10**^£^** | NSF&V | 55 |

***** -% change between intervention and control treatment arms

**^£^** -% change between two treatment arms.

**^@^**-ICC- Intra-class correlation

**^♯^**-SD- Standard deviation

**^**-Primary outcomes are MVPA and NSF&V

**^¢-^** Mean (and standard deviation) minutes of moderate to vigorous physical activity per day of 63.4(37.4)^3^

**^§^**- Mean (and standard deviation) number of daily servings of fruits and vegetables of 4.0(2.5)^4^

**MVPA**-Minutes of moderate to vigorous physical activity per day

**NSF&V**- Number of servings of fruits and vegetables per day

All calculations were made using a 5% level of significance, 80% power and 25 participants per cluster.

1. US Department of Health and Human Services. NIH Research Methods Resources. Sample size calculator for group randomized trials. <https://researchmethodsresources.nih.gov/SampleSizeCalculator.aspx>. Accessed June 2019
2. Hayes RJ, Moulton LH. Cluster randomised trials. Chapman and Hall/CRC; 2017 Jul 6.
3. Oyeyemi AL, Muhammed S, Oyeyemi AY, Adegoke BO. Patterns of objectively assessed physical activity and sedentary time: Are Nigerian health professional students complying with public health guidelines?. PloS one. 2017 Dec 27;12(12):e0190124.
4. Peltzer K, Phaswana-Mafuya N. Fruit and vegetable intake and associated factors in older adults in South Africa. Global health action. 2012 Dec 1;5(1):18668.
5. Eldridge SM, Chan CL, Campbell MJ, Bond CM, Hopewell S, Thabane L, Lancaster GA. CONSORT 2010 statement: extension to randomised pilot and feasibility trials. Pilot and feasibility studies. 2016 Dec;2(1):64.
